# New Phenotypes of Potato Co-induced by Mismatch Repair Deficiency and Somatic Hybridization

**DOI:** 10.3389/fpls.2019.00003

**Published:** 2019-01-22

**Authors:** Elena Rakosy-Tican, Enikö Lörincz-Besenyei, Imola Molnár, Ramona Thieme, Frank Hartung, Thorben Sprink, Olga Antonova, Ivan Famelaer, Geert Angenon, Adriana Aurori

**Affiliations:** ^1^Plant Genetic Engineering Group, Faculty of Biology and Geology, Babeş-Bolyai University, Cluj-Napoca, Romania; ^2^Institute for Biosafety in Plant Biotechnology, Julius Kühn-Institut, Quedlinburg, Germany; ^3^Institute for Breeding Research on Agricultural Crops, Julius Kühn-Institut, Quedlinburg, Germany; ^4^Department of Plant Biotechnology, N.I. Vavilov Institute of Plant Industry, St. Petersburg, Russia; ^5^Laboratory of Plant Genetics, Vrije Universiteit Brussel, Brussels, Belgium

**Keywords:** antisense strategy, *AtMSH2* gene, dominant negative mutant, microsatellite instability, mismatch repair deficiency, ‘mutator’ phenotype, *Solanum chacoense* Bitt

## Abstract

As plants are sessile they need a very efficient system for repairing damage done by external or internal mutagens to their DNA. Mismatch repair (MMR) is one of the systems that maintain genome integrity and prevent homeologous recombination. In all eukaryotes mismatches are recognized by evolutionary conserved MSH proteins often acting as heterodimers, the constant component of which is MSH2. Changes affecting the function of *MSH2* gene may induce a ‘mutator’ phenotype and microsatellite instability (MSI), as is demonstrated in *MSH2* knock-out and silenced lines of *Arabidopsis thaliana*. The goal of this study was to screen for ‘mutator’ phenotypes in somatic hybrids between potato cvs. ‘Delikat’ and ‘Désirée’ and MMR deficient *Solanum chacoense* transformed using antisense (AS) or dominant negative mutant (DN) *AtMSH*2 genes. The results demonstrate that first generation fusion hybrids have a range of morphological abnormalities caused by uniparental MMR deficiency; these mutant phenotypes include: dwarf or gigantic plants; bushiness; curled, small, large or abnormal leaves; a deterioration in chloroplast structure; small deep-purple tubers and early dehiscent flowers. Forty percent of the viable somatic hybrids planted in a greenhouse, (10 out of 25 genotypes) had mutant phenotypes accompanied by MSI. The majority of the hybrids with ‘mutator’ phenotypes cultured on media containing kanamycin developed roots so sustaining the presence of selectable marker gene *npt*II, from the initial constructs. Here for the first time, MMR deficiency combined with somatic hybridization, are used to induce new phenotypes in plants, which supports the role of MMR deficiency in increasing introgressions between two related species.

## Introduction

Mismatch repair (MMR) is a highly conserved mechanism responsible for maintaining genome stability. The MMR system in plants and other eukaryotes involves many proteins that recognize, excise, and repair the DNA mismatches that occur during DNA replication or due to damage (Bray and West, 2005). The key protein in the MMR system in plants and other eukaryotes is MSH2, which forms heterodimers with other MSH proteins. These heterodimers recognize mismatches and interact to initiate repair. The MSH2–MSH6 heterodimer (MutSα) recognizes base-base mismatches and small insertion/deletion loops (IDLs), while MSH2–MSH3 (MutSβ) repairs relatively large IDLs (Culligan and Hays, 2000; Tian et al., 2009). MSH2–MSH7 (MutSγ) preferentially repairs base-base mismatches (Gómez and Spampinato, 2013). Recent data indicate that in eukaryotes only 10–15% of MMR events are directly associated with replication and MutSα is able to scan genomes independently. How it does this is unclear (Hombauer et al., 2011). Mismatched bases can also arise from homeologous recombination. In contrast to homologous recombination that occurs during meiosis in complementary DNA sequences, homeologous recombination is an illegitimate recombination. It involves recombination of divergent DNA sequences with similar but not identical DNA molecules. MMR proteins are involved in the suppression of homeologous recombination in *E. coli* and *Salmonella typhimurium* (Harfe and Jinks-Robertson, 2000). Defective MMR generally causes the occurrence of a ‘mutator’ phenotype, which is characterized by an accumulation of random mutations in the genome. In plants, phenotypic mutations and microsatellite instability (MSI) are associated with MMR deficiency (Leonard et al., 2003; Hoffman et al., 2004; Chao et al., 2005; Spampinato et al., 2009; Xu et al., 2012). Moreover, more recent data indicates that in hybrid rice introgressions from the wild species *Zizania latifolia* induce microsatellite instability, alter MMR activity and result in novel phenotypes (Dong et al., 2013).

The *MSH*2 gene was first sequenced in the model species of plant, *Arabidopsis thaliana* (Culligan and Hays, 1997; Adé et al., 1999). In *A. thaliana*, the inactivity of *MSH2* and *PMS1* results in an increase in homeologous somatic (mitotic) recombination when the sequences vary between 0.5 and 9% (Li et al., 2006, 2009). Mutants of *MSH2* induce a threefold increase in intrachromosomal recombination in germinal tissues of *A. thaliana* between highly diverged sequences (13%) (Lafleuriel et al., 2007). DNA sequence analysis of subsequent generations of MMR-deficient yeast reveal a threefold increase in mutation rate and random genome-wide distribution of mutations. MSI is more frequent in homopolymeric poly A or T genomic stretches (Lang et al., 2013). MMR plays an important role in gene stability as genes are more prone to mutations in *A. thaliana* with defective MMR. Intergenic regions are less affected by mutations (Belfield et al., 2018). The effect of defective *AtMSH2* indicates the role this gene has in maintaining germline stability and its role in somatic cells as less critical (Hoffman et al., 2004). A better understanding of how plants control meiotic or somatic (mitotic) recombination could improve the breeding of superior varieties, particularly when this involves exchange of genetic material between related species (Li et al., 2006; Martinez-Perez and Moore, 2008; Wijnker and de Jong, 2008). Such an exchange of genes is expected in somatic hybrids between the two related species of potato *Solanum tuberosum* and *S. chacoense*.

The genetic diversity of many crop plants, including potato, which ranks fourth in world-wide productivity, has been depleted by continuous cultivation. The wealth of wild *Solanum* tuber-bearing species (∼226), which are closely related to cultivated potato, are an important reservoir of resistance genes for potato improvement (Hawkes, 1990). This is a source that might be used in combinatorial biotechnology, e.g., for breeding resistance (Rakosy-Tican, 2012; Rakosy-Tican et al., 2016; Thieme and Rakosy-Tican, 2017). *Solanum chacoense* Bitt. (*chc*) is a highly polymorphic, tuber-bearing diploid species (2n = 2x = 24) with a more divergent germplasm pool than *Solanum tuberosum*. *Chc* is a weed in lowland pastures in South America. This species is resistant to insects [(Colorado potato beetle (CPB) -*Leptinotarsa decemlineata*) (Sinden et al., 1986)], fungi [*Verticillium* wilt (Lynch et al., 1997)], bacterial diseases [common scab and soft rot (Simko et al., 2007)], viruses [potato virus X (PVX), potato virus Y (PVY) (Cockerham, 1970; Sato et al., 2006), potato leaf roll virus (PRLV) (Brown and Thomas, 1994)], root-knot nematodes (Janssen et al., 1996) and is more tolerant of heat and drought stress (Hanneman and Bamberg, 1986). Hence, *chc* is a valuable species for improving potato cultivars, either by crossing them sexually or somatic hybridization (Cheng et al., 1995). Although it is possible to cross potato and *chc*, such classical breeding takes a long time and has only resulted in a few potato lines with some resistance to CPB (Thompson et al., 2008).

In previous research, there are reports of transgenic MMR deficient clones of the high leptine producing accession of *S. chacoense* (PI 458310), with either an antisense (AS) or a dominant negative (DN) mutant of the *AtMSH2* gene (Rakosy-Tican et al., 2004). The growth of roots and plants on MS media containing kanamycin and RT-PCR analysis has confirmed the transgenic status of some of these clones. The hypothesis tested in this study is that one MMR-deficient parent will increase the number of mutant phenotypes and incidence of homeologous recombination in the resultant somatic hybrids. This is why three transgenic MMR-deficient clones were selected for mesophyll protoplast isolation and fusion with the potato cultivars ‘Delikat’ and ‘Désirée.’ The goals of this study are to: (1) identify abnormal phenotypes in somatic hybrids (SHs) involving MMR-deficient or wild type *S. chacoense, in vitro* and *ex vitro*; (2) differentiate between ‘mutator’ phenotypes induced by MMR-deficiency and other abnormal phenotypes caused by somatic fusion or *in vitro* culture; (3) discuss the role of increased homeologous recombination in inducing the introgression of resistance traits into the potato gene pool; (4) establish the importance of phenotypic variability co-generated by MMR deficiency and somatic hybridization in a wider context by using different tools for breeding potato with many resistant traits.

## Materials and Methods

### Plant Material and Selection of Transgenic Clones of *Solanum chacoense* Deficient in MMR

**FIGURE 1:**
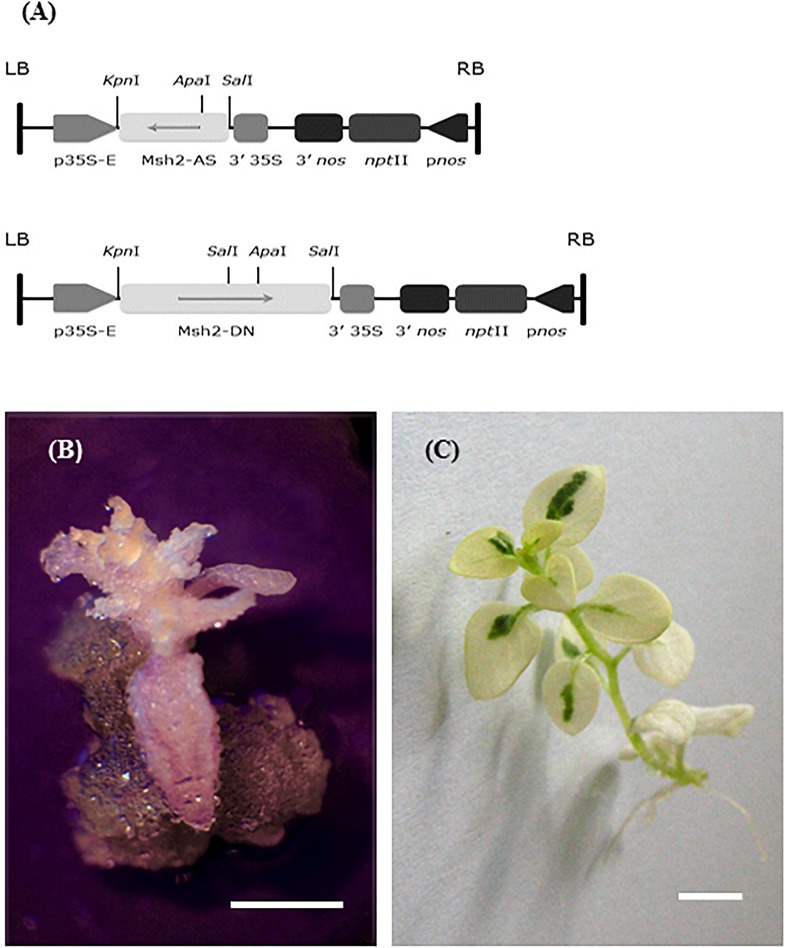
Schematic representation of the constructs transformed into *Solanum chacoense* to induce MMR deficiency. **(A)** T-DNA of the FRG-*MSH*2-AS plasmid (up) and T-DNA of the FRG-MSH2-DN plasmid (down – Ispas, 2004); two mutant shoots that regenerated *in vitro*. **(B)** MMR deficient transgenic DN *S. chacoense* HL, callus on principal root of an albino shoot (callus-like albino shoot). **(C)** Somatic hybrid DkAS – chimeric albino mutant phenotype; bar = 1 cm.

Seeds of *Solanum chacoense* Bitt. (*chc*), accession PI 458310, known as a high leptine producer (HL), were provided by NPGS, Sturgeon Bay, WI, United States. The seeds were sterilized by washing in 70% ethanol for 1 min, followed by 7% (v/v) Domestos (commercial bleach which contains about 5% sodium hypochlorite) for 20 min, then rinsed three times with sterile water and aseptically germinated on MS ½ salts medium (Murashige and Skoog, 1962). *In vitro* regenerated plants (on RMB5, i.e., MS salts with vitamin B5 medium, Duchefa) were used for genetic transformation. The genetic transformation of *chc* was done using *A. tumefaciens* LBA 4404 (Rakosy-Tican et al., 2004). The constructs for genetic transformation are presented in Figure 1. The AS construct contains the 3′1 kb fragment of the *AtMSH*2 cDNA in antisense orientation. The DN construct contains the *AtMSH2* coding sequence with a mutation converting the strongly conserved Gly codon at position 697 into an Asp codon (Ispas, 2004). The same mutation at a homologous position in the yeast *MSH2* gene confers a strong dominant negative phenotype (Nicolaides et al., 1998; Studamire et al., 1999). The putative transgenic AS and DN clones were tested both phenotypically (growth of roots and callus on 50 mg/L kanamycin medium) and by RT-PCR (Rakosy-Tican et al., 2004). The transgenic clones were screened for potential mutations *in vitro* and *ex vitro*. One transgenic line for AS and two for DN, respectively, without any phenotype abnormality (see Supplementary Table S1), and two cultivars of potato, ‘Delikat’ (Nordring-Kartoffelzucht – und Vermerungs – GmbH Gross Lüsewitz, Germany) and ‘Désirée’ (ZPC, Leeuwarden, Netherlands) were propagated *in vitro* on RMB5 medium (21°C ± 2°C; photoperiod 16:8 h; fluorescent light intensity of 90 μmol m^-2^ s^-1^) and used for mesophyll protoplast isolation (3- to 4-week-old *in vitro* plants).

### Protoplast Isolation, Electrofusion, and Culture of Fusion Products

The protocols for isolating mesophyll protoplasts, electrofusion and culture were mainly as described previously (Thieme et al., 2008). Electrofusion was carried out using a home-made generator and an adapted electroporation cuvette. The best parameters were determined by using a microscope slide with gold electrodes attached to the glass using photolithography and a gap of 500 μm between the electrodes. After establishing the right parameters, electrofusion was carried out in an electroporation cuvette with a width of 2 mm (Sigma-Aldrich^®^). Electrofusion parameters were: dielectrophoresis at 1 MHz and 800 V; electrofusion with two square pulses of 2000 V cm^-1^ of 30 μs duration. In order to allow the fusion products to stabilize, the suspension of protoplasts was pipetted out of the cuvette 30 min post-electrofusion. The suspension made up of 1:1 protoplast species at a final density of 1 × 10^6^ protoplasts ml^-1^ was mixed with culture medium and cultured as described by Thieme et al. (2008, 2010). Plating efficiency, i.e., the number of colonies of cells regenerated from 100 plated protoplasts was determined after 2 weeks by examining the plates under a CK2 inverted microscope (Olympus Europa GmbH Hamburg, Germany). At the level of a colony of cells a more detailed comparative assessment was undertaken in terms of the diameter of the colony and the cells, and the number of cells per colony. This was done by examining the cultures under an Olympus inverted CK2 microscope using an ocular micrometer, in five to ten randomly selected microscope fields. The first shoots to be regenerated by each callus were selected and cultured, based on the assumption of vigorous growth of interspecific fusion products (Rakosy-Tican and Aurori, 2015). *In vitro* grown plants were used for further analysis. The parents and somatic hybrids (SHs) were also maintained for medium-term *in vitro* as micro-tubers on MS medium supplemented with 10% sucrose, at 4°C.

### Ploidy Analysis

Putative hybrid plants were screened for ploidy by using flow cytometry (Thieme et al., 2008). To estimate the stability of the level of ploidy after long-term culture *in vitro* and storage as micro-tubers for almost 7 years, the ploidy of *in vitro* clones was checked again by using the protocol described by Doležel et al. (2007) and staining the DNA with propidium iodide (see Rakosy-Tican et al., 2015).

### Assessment of Morphological Abnormalities *in vitro* and *ex vitro*

The regenerated transgenic lines of *S. chacoense* Bitt accession PI 458310 and SHs with potato commercial cultivars ‘Delikat’ and ‘Désirée’ were assessed, by comparison with respective wild types, for morphological abnormalities both *in vitro* and *ex vitro*. The wild type SHs are coded using numbers (i.e., SH 1837/1) or C followed by a number (control, i.e., DkC 5; DeC 7). The potato cultivars are designated as Dk = ‘Delikat’ and De = ‘Désirée.’ When the dominant negative mutant *AtMSH2* gene is present in the *chc* parent, the SHs are referred to as: DkDN 5, DkDN 11 or DeDN 5 versus DeDN 11, with the numbers followed by the number of each clone. When the AS gene is present then DkAS 10 is followed by the clone number. For cv. ‘Désirée’ none of the regenerated SHs had the AS *AtMSH2* gene (Table 1).

**Table 1:** The viable somatic hybrids and transgenic clones with MMR deficiency propagated *in vitro*, maintained as micro tubers and transferred to a greenhouse (*n* = 25 SHs).

*Solanum chacoense* HL transgenic clones and controls	Somatic hybrids DkDN	Somatic hybrids DeDN	Somatic hybrids DkAS
*chc* DN 5	DkDN 5.3	DeDN 5.5	DkAS 10.5
*chc* DN 11	DkDN 5.4	DeDN 11.5	DkAS 10.8
DkC 5	DkDN 5.6	DeDN 11.29	DkAS 10.11
DkC 7	DkDN 5.7		DkAS 10.13
DeC 7	DkDN 5.11		DkAS 10.20
DeC 8	DkDN 5.17		DkAS 10.35
1837/1 (control, wild type – WT)	DkDN 5.25		DkAS 10.40
1552/1 (control WT)	DkDN 11.10		DkAS 10.43
1913/6 (control WT)	DkDN 11.24		DkAS 10.47
1913/10 (control WT)	DkDN 11.26		DkAS 10.51
	DkDN 11.34		DkAS 10.61


The plants were acclimatized *ex vitro* planted in pots filled with good quality garden soil and covered with a plastic beaker in a greenhouse for 2 weeks. The light source in the green-house was natural, with a photoperiod of 16:8 h and a temperature varying between night and day (14–16°C during night and 22–25°C, during the day). The height of the plants, leaf and internode length were measured in cm when the plants were 61 days old (2 months after the end of vegetative growth). Mean values and standard deviations were calculated for at least seven clones per genotype (*n* = 7). Photographs that were taken of all the plants and their leaves, flowers and flower development were analyzed. At the end of vegetative growth tubers were collected, weighed and photographed (Olympus digital camera 5060). The color and number of tubers were also evaluated. For the comparison of the tubers photographs of the two biggest and three smallest tubers per seven plants are in the Supplementary Figure S3.

### Correlation Between Plant Height and Ploidy Level

The height of the plants was measured from soil level up to the apical meristem (*n* = 7), and the relationship between plant height and ploidy was described by a linear regression, in order to identify SHs with a ‘mutator’ phenotype. This evaluation was based first on flow cytometry data of the diploid *chc* parent, tetraploid potato parents and one hexaploid SH as well as on the presumption that plant height increases with ploidy level (Mizukami, 2001).

### Assessment of Drought Tolerance and Colorado Potato Beetle Resistance

Drought stress was assessed *in vitro* by adding 5% or 15% PEG 6000 to micro propagation media. The growth of the plants and development of their roots were recorded, and plants were classified as tolerant or sensitive to drought depending on their growth. Five clones were analyzed for each genotype and the experiments repeated twice. Parental plants were compared to somatic hybrids without (controls) or with a MMR-deficiency. Then the plants that were considered to be tolerant and some sensitive and parental lines, were transferred to a phenotyping platform (Biological Research Centre, Szeged, Hungary) and after determining their biomass and photosynthesis were confirmed as tolerant or sensitive to a 20% water content in the soil (data prepared for another publication). In this study, only the final results for mutant phenotypes are indicated.

A laboratory bioassay and a choice test were used to evaluate the level of antibiosis and antixenosis, respectively, for CPBs as previously reported (Molnár et al., 2016). Data on drought tolerance and resistance to CPB are presented in Table 2 as indicators of the possible practical applications of mutagenesis induced by both MMR deficiency and somatic hybridization.

**Table 2:** Type of mutation, ploidy level, SSR instability, flowering and root development on media containing kanamycin as well as tolerance to drought and resistance to Colorado potato beetle in somatic hybrids between potato + MMR deficient *Solanum chacoense* that produced at least one mutant phenotype in a greenhouse; ND, not determined; +, yes; -, no.

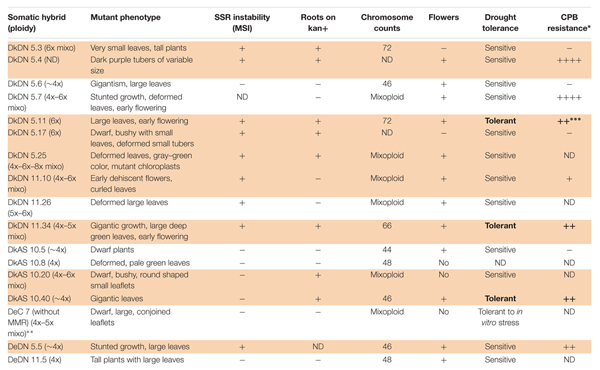					

*^∗^Resistance to Colorado potato beetle (CPB) was evaluated as indicated by Molnár et al. (2016), and is presented here as: ++++, both highly antibiotic and antixenotic, like the wild parent; ++, antibiosis and antixenosis intermediate between that of the parents; +, only antibiotic to CPB; -, lack of both resistances against CPB. ^∗∗^Used as control – leaf abnormalities might be caused by somatic fusion. ^∗∗∗^The somatic hybrids with both traits, resistance to Colorado potato beetle and tolerance to drought are highlighted in bold; ‘mutator’ genotypes are highlighted in pale-red.*

### Molecular Analysis – Microsatellite Instability

DNA was isolated from *in vitro* plants of the SHs *S. tuberosum* + *chc* with and without a MMR deficiency, as well as their parents. DNA was extracted from leaves using the protocol described by Lodhi et al. (1994), modified by Pop et al., 2003). For revealing MSI 96 SSR markers were used, the majority of which are chromosome specific (Supplementary Table S5). Two of them are illustrated in Figures 6A,B, Sti 046 and Sti 054, which are on chromosomes 11 and 12, respectively (Feingold et al., 2005). A 16-bp extension TGT AAA ACG ACG GCC AG, M13 primer, was added to the 5′-end of the forward primer. PCR reactions were carried out in 12 μL covered with layer of mineral oil in a Biometra Uno II thermocycler (Whatman Biometra, Göttingen, Germany). For each assay, 50 ng of genomic DNA in a solution containing 1 × reaction buffer, 2.5 mM MgCl_2_, (Qiagen, Hilden, Germany), 0.6 mM dNTP, 0.25 mM of each primer, 0.15 pmol of an IRD800-labeled M13 sequencing primer and 0.25 U HotStarTaq DNA polymerase (Qiagen) was used. Thermal cycling conditions were: (1) 94°C for 14 min and 15 s (2) six cycles at 94°C for 45 s, 57°C for 1 min, 72°C for 1 min, (3) 30 cycles at 94°C for 45 s, 54°C for 45 s, 72°C for 1 min, (4) 72°C for 5 min. Conditions for electrophoresis: prior to electrophoresis, 8 μL of loading buffer (10 mM EDTA, 0.1% basic fuchsine, 0.01% bromophenol blue, 95% formamide) was added to each sample; samples were denatured for 5 min at 95°C with a subsequent application of 1 μL to the gel. Gel electrophoresis and pattern visualization were carried out on LI-COR Model 4300 automated fluorescent DNA sequencer (LI-COR Inc., Lincoln, NE, United States) using a polyacrylamide gel. The gel contained 6% Long Ranger gel solution (BMA, Biowhittaker Molecular Applications, Rockland, ME, United States), 10% ammonium persulfate (APS) and tetramethylethylenediamine. Gels were run in a modified 1 × TBE running-buffer (134 mM Tris, pH 8.3, 44 mM boric acid, 2.5 mM EDTA).

### Root Development on a Medium Containing Kanamycin

Nodal segments of different SHs (including wild types, controls) and both parents were placed on a RMB5 medium containing 50 mg L^-1^ kanamycin. After 20 and 30 days the development of the roots was assessed by measuring the number and lengths of the roots. This was done five times (*n* = 5) and repeated twice.

### RT-qPCR Analysis of the *MSH2* Gene

Plants propagated *in vitro* on MS medium for 2 weeks, including parents and SHs with or without MMR deficiency, were acclimatized in a greenhouse. After an additional 2 weeks, the samples were collected for isolation of RNA. The environmental conditions were 20°C and a 16/8 h photoperiod. The hybrid status of SHs was previously confirmed by SSR marker analysis. Total RNA was isolated using the Bio and Sell RNA mini Kit (Bio&Sell e.K., Feucht, Germany) following the manufacturer’s instructions. To evaluate the abundance of *MSH2* at the somatic level, leaf material was used. To avoid contamination of genomic DNA, isolated RNA was treated with RNase free DNase I (Thermo Fischer Scientific, Germany). To inhibit RNase activity RiboLock RNase inhibitor (Thermo Fischer Scientific, Germany) was used. RNA was purified and concentrated using a Gene JET RNA Purification Kit (Thermo Fischer Scientific, Germany). Agarose gel electrophoresis (1.2%) and spectroscopy were used to confirm the integrity and quality of the RNA. Reverse transcription was performed using an anchored oligo (dT)_18_ Primer and the Maxima H Minus First Strand cDNA Synthesis Kit (Thermo Fischer Scientific, Germany) using 5 μg of total RNA as a template for the RT-reaction. The qPCR reactions were done using Maxima SYBR Green qPCR Master Mix (2x) (Thermo Fischer Scientific, Germany) in a BIO-RAD CFX96 Real-Time PCR Detection System (Bio-Rad, Hercules, CA, United States). As a reference gene, protein phosphatase 2A (PP2A) was used based on results of studies on potato (Katsarou et al., 2016) and *Nicotiana benthamiana* (Liu et al., 2012). The primers were designed manually using NCBI Primer Blast to span exon–exon junctions^1^. RT-qPCR primers used were for *MSH2* (NCBI Acc. XM_006354671.2): forward 5′-GTTTCTCCGAGGTGTTTGAAGGT-3′, reverse 3′-CTCCCGTATCTGGTGGACTG-5′; for *PP2A* (NCBI Acc. XM_006362495.2) forward 5′-CTAAGGATAGGGTGCCCAAC-3′ and reverse 3′-TCTCCACCACCGAGTTGTC-5′.

PCR amplification was done using the following program: 95°C for 10 min followed by 44 cycles of 95°C for 10 s, 61°C for 20 s, 72°C for 30 s and 95°C for 1 min and cooling to 55°C for 30 s, finally a melting curve was generated, which ranged from 55°C to 95°C in increments of 0.5°C. Three biological and three technical replicates were used and in each run the reference gene was amplified in order to avoid differences between the runs. For data analysis mean Cq values of the 10-fold dilution series were plotted against the logarithm of the pooled cDNA dilution factors. The Cq values were used to determine the efficiency (E) of each gene based on the slope of a linear regression model with the following equation: % E = (10^[-1/slope]^- 1) × 100% (Radonić et al., 2004). *R*^2^ values were taken into consideration in all the situations in which the *R*-value was between 0.990 and 0.995. The efficiency of all PCR amplifications was between 90% and 110%. All the biological replicates were used to calculate the average Cq value. Fold change relative to the expression of the sample genes and reference genes was calculated using the normalized expression [ΔΔ(Ct)] method (Livak and Schmittgen, 2001), with default threshold values, using CFX Manager Software^TM^ (Bio-Rad, Hercules, CA, United States).

### Data Statistical Analysis

For the linear regression analysis and Student’s *T*-test the XLSTAT and R statistical software version 3.3.1 were used. In the analysis *p*-values less than 0.05 were considered statistically significant.

## Results

### Selection and Evaluation of Transgenic MMR Deficient Clones

In order to evaluate the effects of MMR deficiency on somatic hybridization it was first important to select the transgenic accession of *chc* high in leptine carrying the two constructs (Figure 1A) and to confirm the transgenic status of the clones used for protoplast isolation. Out of total 29 putative transgenic plants resulting from the *Agrobacterium*-mediated transfer of *AtMSH*2 in antisense orientation (AS-15) and *AtMSH*2 dominant negative (DN-14) clones, only five and three plants, respectively, were positive in RT-PCR tests (Supplementary Table S1). Three transgenic lines, denoted AS 10, DN 5 and DN 11, which grew on the medium containing kanamycin and positive in the RT-PCR, were chosen for protoplast isolation.

### Regeneration of Plants and Assessment of Level of Ploidy

#### The Efficiency With Which Somatic Hybrid Plants Can Be Regenerated

The efficiency with which mesophyll protoplasts can be isolated is similar for all fusion partners, i.e., potato cultivars ‘Delikat’ and ‘Désirée,’ *chc* wild type and the three transgenic MMR deficient clones, with values of approximately 1 × 10^6^ pp mL^-1^ g*^-^*^1^ mesophyll tissue. Thus it was possible to carry out electrofusion in large volumes after evaluation of the best parameters based on the studies using a microscope slide (Supplementary Figure S1). Assessment of plating efficiency and regeneration of first shoots was done by comparing wild type and transgenic AS and DN *chc* when fused with potato cv. ‘Delikat,’ This revealed that plating efficiency, calculated as the % of cell colonies that developed from plated mesophyll protoplasts, for the fusion combination involving potato and wild type *chc* was two and four times greater than that for *chc* DN and AS, respectively (Supplementary Table S2). In contrast, plant regeneration was better for transgenic MMR deficient *chc*, with five and four times more regenerates for DN and AS fusion hybrids, respectively. Moreover, cv. ‘Delikat’ produced more regenerated shoots than the cv. ‘Désirée’ (Supplementary Table S3). In order to understand why more colonies from fusion combinations involving transgenic MMR deficient *chc* were able to regenerate shoots and plants a more detailed analysis of cell colony regeneration was undertaken, which involved comparing the results for potato cv. ‘Delikat’ and *chc* AS with those for the wild type SHs and potato protoplasts (Supplementary Figure S2). Interestingly, there were no significant differences between the mean diameters of cell colonies, although the colonies from wild species are less compact than those from *chc* with the AS *AtMSH*2 gene (Supplementary Figure S2A). In addition, there were significant differences between the mean numbers of cells per colony, with the wild type having fewer cells. Moreover, there were highly significant differences in cell diameter with the SH involving transgenic AS *chc* colonies having larger cells than in those of hybrids involving wild type *chc* (Supplementary Figures S2B,C). The wild type *chc* protoplasts did not divide in the same way as those that were fusion products and were unable to regenerate plants. Features of these cell colonies differed from those regenerated from unfused control protoplasts of potato cv. ‘Delikat’ (Supplementary Figure S2D1). The external cells of colonies derived from fusion products are long and skittle-shaped, whereas those derived from transgenic *chc* AS are larger (Supplementary Figures S2D2, D3 – diameter measured across the longest axis). During development of the micro-calluses the skittle-shaped cells divide transversally many times and produce isodiametric cells. These interesting differences might account for the above differences in plant regeneration (Supplementary Figure S2).

#### Assessment of Ploidy Level

**FIGURE 2:**
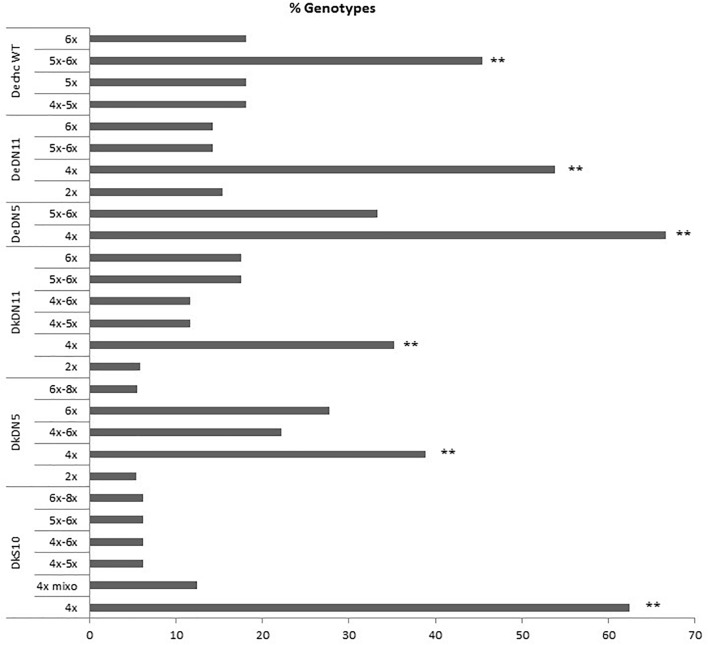
Ploidy of the somatic hybrids, comparison of the ploidy levels (%) of different fusion combinations for potato ‘Delikat’ (Dk) and ‘Désirée’ (De) with *Solanum chacoense (chc)* HL wild type (WT) or MMR deficient clones (AS –*ATMSH2* gene in antisense orientation; DN – dominant negative *ATMSH2* gene, numbers indicate the somatic hybrid clone); mixo – mixoploid; ^∗∗^ significant at *p* < 0.05 after pairwise comparison of groups of ploidy as compared to wild type groups (5x–6x+6x/6x or 4x+4x–5x/4x).

Flow cytometry revealed the ploidy level of the parental genotypes and derived SHs, which are in Supplementary Table S4. When the different ploidy levels of the wild type somatic hybrids and those with MMR deficient *chc* are compared the preponderance of ploidy levels is striking. For the combination involving wild type *chc* the majority of SHs have 5x–6x or 6x levels of ploidy as theoretically expected, for those involving MMR deficient *chc*, either AS or DN, the majority of the SHs are tetraploids, near tetraploids or mixoploids (Figure 2 and Table 2). The ploidy level of some of the somatic hybrids was reassessed after years of *in vitro* culture and storing as micro-tubers (Supplementary Table S4). The results of which indicate that the SHs are genetically stable as no changes in ploidy level were recorded. Moreover, what seem to be tetraploid SHs are in fact mainly aneuploids, as shown by the chromosome counts (Table 2), which may indicate that MMR deficiency may have an effect on the elimination of chromosomes and cause an increase in introgressions (data under investigation using molecular cytogenetics).

### Analysis of Regenerated Somatic Hybrid Clones

#### Identification of ‘Mutator’ Phenotypes *in vitro* and *ex vitro*

Among the few transgenic AS and DN clones of *chc* there was one mutant phenotype in the *in vitro* culture, a callus-like shoot (Figure 1B). During the *in vitro* stage of regeneration of somatic hybrid clones with MMR deficiency one chimeric albino occurred, which denotes a mutation in chlorophyll biosynthesis (Figure 1C). These defective, ‘mutator’ phenotypes were lost during *in vitro* multiplication.

**FIGURE 3:**
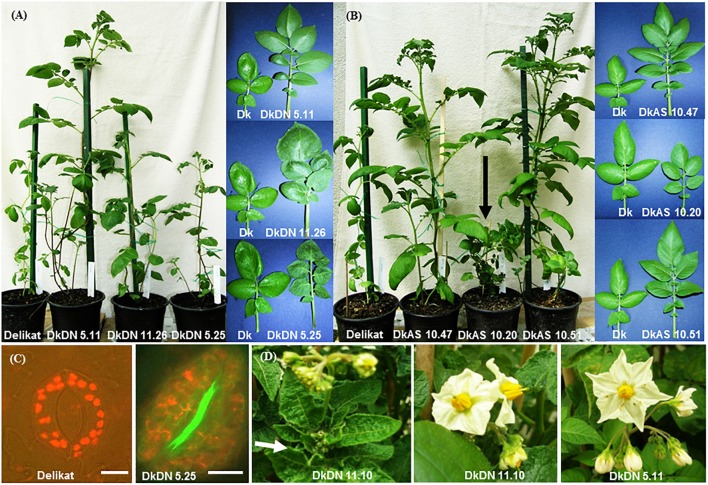
Comparison of the phenotypes of the somatic hybrids with potato parent ‘Delikat’ and photographs of whole plants, leaves, and a mutant flower. **(A)** Phenotypes of the plants grown in a greenhouse (61 days), from left to right: potato cv. ‘Delikat,’ and ‘mutator’ phenotype of the SHs with MMR deficiency as indicated; on the right comparison of the morphology of the leaves of the same SHs and cv. (Dk). **(B)** Phenotype of whole plants and morphology of the leaves of the SHs of the same age grown in a greenhouse, compared to those of cv. ‘Delikat’: whole plants of SHs involving transgenic *chc* with antisense *Atmsh2* gene, (DkAS 10.20 dwarf mutant-black arrow). Note the small leaf with round-shaped folioles of SH DkAS 10.20 with a dwarf ‘mutator’ phenotype, which is unlike the large leaves of the other two SHs. **(C)** Shows details of the chloroplasts in the guard cells of potato cv. ‘Delikat’ and mutant SH DkDN 5.25 (scale bar = 10 μm) (see leaf phenotype in **A**). **(D)** Mutant with early opening flower buds and curled leaves (arrow) in the somatic hybrid DkDN 11.10 (left buds and right mature flower), compared to SH DkDN 5.11, with a normal flower phenotype, bar = 1 cm.

**FIGURE 4:**
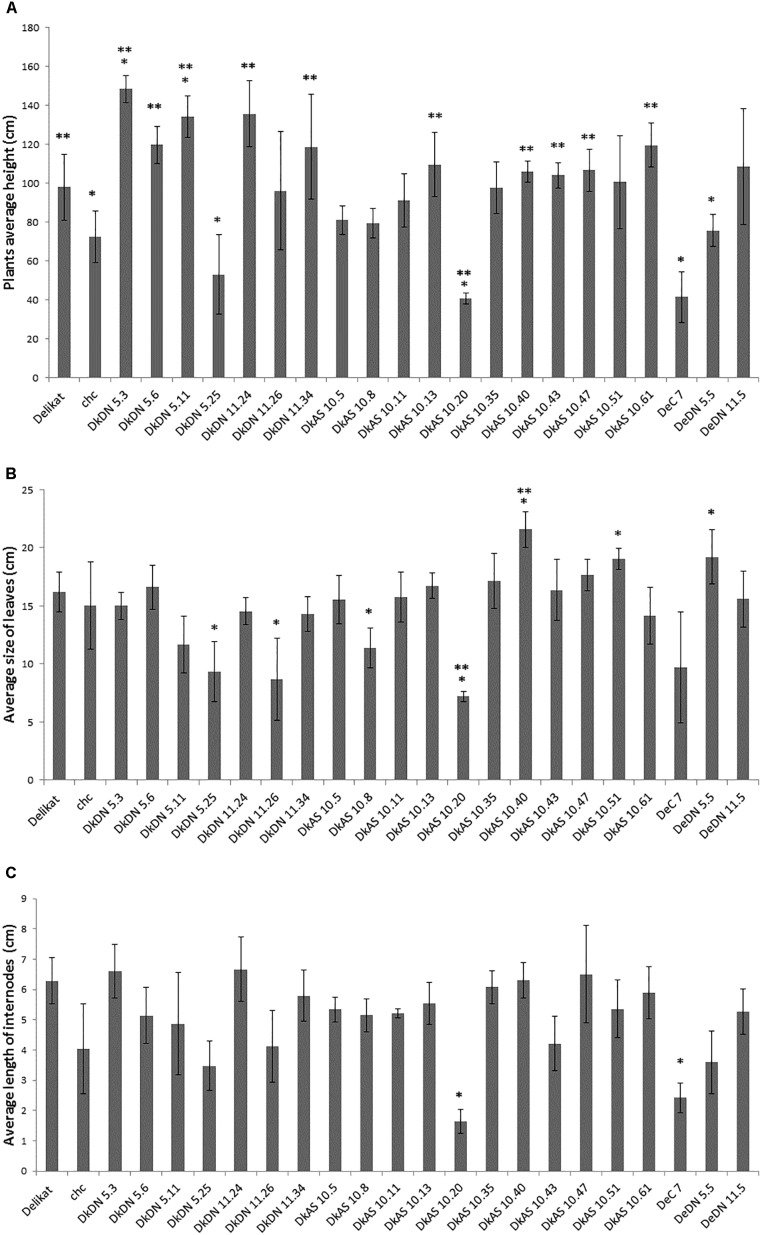
Biometric data of the somatic hybrids of potato + *Solanum chacoense*, with or without MMR deficiency grown in a greenhouse for 61 days after transfer from *ex vitro.* **(A)** Average height of the plants (cm). **(B)** Average size of the leaves – length along midrib (cm). **(C)** Average length of internodes (cm). ^∗^ Significantly different from *S. tuberosum* at *p* < 0.005; ^∗∗^ significantly different from *S. chacoense* at *p* < 0.005; bars = SD.

**FIGURE 5:**
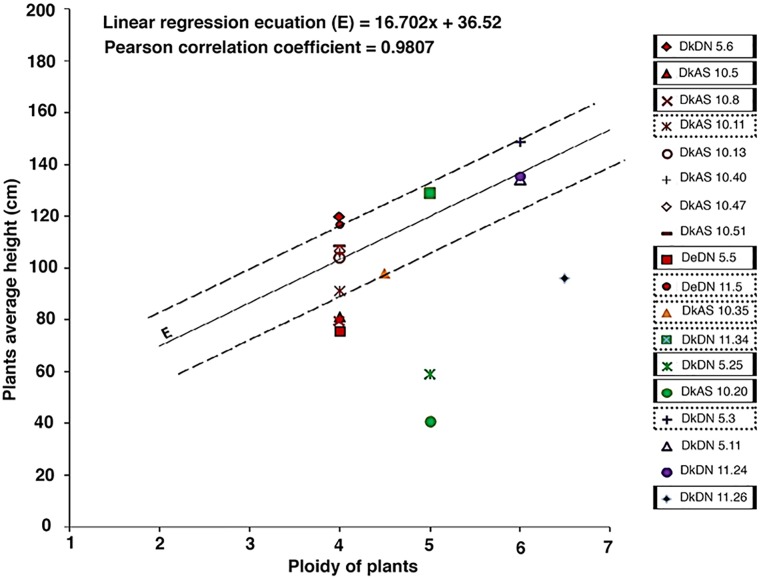
The correlation between plant height and ploidy level (based on flow cytometry data), genotypes that differ significantly (*p* < 0.05) from that predicted by the linear regression are considered to have an abnormal phenotype (dotted line rectangles), while those far from linear line (*p* < 0.01) are considered to be ‘mutator’ phenotypes (full line rectangles).

The phenotypic analysis of the hybrids between *chc* AS and potato ‘Delikat’ that were transferred and grown in soil in a greenhouse revealed that they were very variable in height with some that were stunted (DkAS 10.20), i.e., a dwarf phenotype (Figure 3B and Table 2). Among the hybrids between transgenic DN and AS *chc*, there were gigantic plants and plants with very large leaves (Figures 3A,B, Table 2, and Supplementary Figure S3B). The third leaf down from apex of some of the SHs was abnormal compared with that of their wild parents (Table 2, Figure 3, and Supplementary Figure S3B). In addition, a slightly different phenotype, with small conjoint leaves, was recorded in the wild type SH DeC 7, a mixoploid between *chc* and potato cv. ‘Désirée’ (Table 2). Later on, a mutant with abnormal flower development was identified in the culture room, in which the flower buds dehisced early and the sepals and petals were small, features that were less evident when the flower was completely open – DkDN 11.10 (Figure 3D and Supplementary Figure S3A). This mutant clone also had large curled leaves (Figure 3D and Table 2). Among the somatic hybrids between DN clones there were some with deformed or very large leaves and flowers that were slightly different in shape (Table 2; Figure 3, and Supplementary Figure S3). Another abnormality in the form of disorganized grana and changes in the structure of the chloroplast in guard cells occurred in SH DkDN 5.25 and was associated with having slightly grayish-green colored and deformed leaves (Figures 3A,D and Table 2). In addition, the inner cell wall around the stoma of the guard cells was thick and a fluorescent green (Figure 3C). When the tubers were compared one SH with MMR deficiency, DkDN 5.4, had variable and very dark purple tubers and another SH DkDN 5.17, had small deformed tubers (Table 2 and Supplementary Figure S3C). In contrast, SHs with AS MMR deficient *chc*, had normal shaped and big tubers when grown in a greenhouse (Supplementary Figure S3C). As for mean tuber fresh weight, those of the SHs: DkDN 5.6, 5.11, 5.25, 11.34, DkAS 10.40 and DeDN 11.5, were all heavier than those of the *S*. *tuberosum* parent, but the number of tubers produced was lower than that produced by both parents (Supplementary Figure S4). The frequency of mutations in first generation somatic hybrids is high when only those that survived when transferred to a greenhouse are considered (25 MMR deficient SHs –Table 1), with at least 40% (10 SHs) with one kind of mutant phenotype associated with MSI (Table 2). The ‘mutator’ phenotypes are confirmed by the data on plant height, leaf size and internode length, measured 61 days after the *ex vitro* transfer (Figure 4). Average length of the internodes varied significantly in only two of the SHs, DkAS 10.20 (dwarf phenotype) and DeC 7 (control SH with wild type *chc*). Leaf size of the SHs, measured along the midrib was significantly different from that of the potato parent in SHs: DkDN 5.25 and 11.26, DkAS 10.8, 10.20 (smaller than potato parent) and DkAS 10.40, 10.51 and DeDN 5.5 (larger than potato parent). A few of the SHs had leaves that were significantly different in length from those of the *chc* parent (the dwarf DkAS 10.20 and gigantic DkAS 10.40) (Figure 4). Somatic hybrids were more variable in plant height than both parental species, which also differed significantly in height. Mean plant height differed significantly in the SHs: DkDN 5.3, 5.6, 5.11, 5.25, 11.24, 11.34, DkAS 10.20, 10.40, 10.43, 10.47, 10.61 and DeDN 5.5. The wild type SH DeC 7 was significantly shorter than potato (Figure 4). The correlation between plant height and ploidy level of the SHs revealed that most of the ‘mutator’ phenotypes differed significantly from that predicted by the linear regression, which confirms their growth was abnormal (Figure 5 and Table 2). Those SHs that were highly significantly different (*p* < 0.01) from that predicted by the linear regression equation also showed MSI and hence were considered as ‘mutator’ phenotypes induced by MMR deficiency (Table 2 – highlighted in red).

#### Tolerance of Drought and Resistance to Colorado Potato Beetle in the Mutant Clones

The data on these traits are published (Molnár et al., 2016) or are prepared for publication (data on drought tolerance). In this paper, only the status of the abnormal phenotypes is included in Table 2. The results indicate that there are SHs tolerant to drought, although both parents were sensitive to this abiotic stress. Consequently, this tolerance is considered to be a novel trait induced by both MMR-deficiency and somatic hybridization. The SH DKAS 10.13, which recovered after being subjected to drought in a greenhouse did not show any other abnormal phenotype. Three SHs were tolerant of drought and resistant to CPB (Table 2 – indicated in bold). As for the antibiotic and antixenotic effects on CPB two SHs listed in Table 2 had traits similar to the wild high leptine parent, another four were intermediate between the parents and one had an antibiotic effect on CPB.

#### Expression of Resistance to Kanamycin in Somatic Hybrids

Since the construct used for transformation also contained the selectable marker gene *npt*II (Figure 1A), its presence enabled SH plants to develop roots on media containing kanamycin. All putative ‘mutator’ clones were evaluated for root growth after 20 and 30 days on media containing kanamycin. With two exceptions, DkDN 11.10 and DkDN 11.26, all ‘mutator’ SH clones with MSI developed roots on media containing kanamycin within 20 days (Table 2).

#### Microsatellite Instability and Molecular Analysis of Hybridity

**FIGURE 6:**
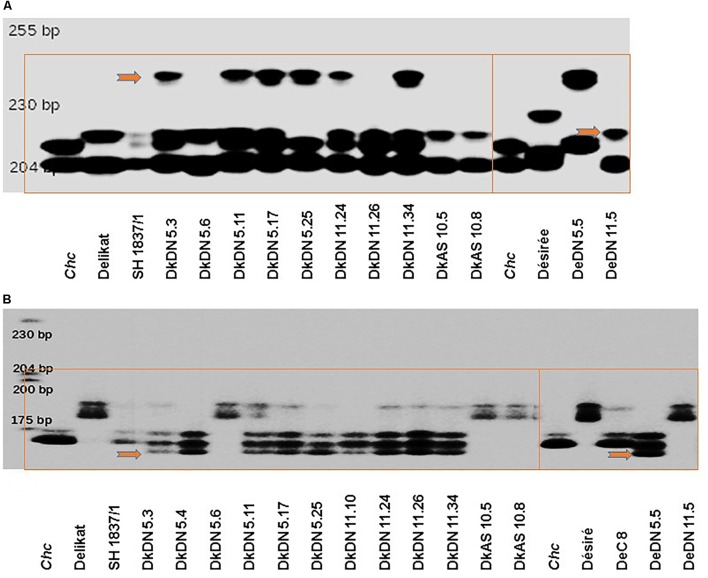
Examples of SSR instability in *Solanum tuberosum* + *S. chacoense* MMR deficient somatic hybrids in comparison with their parents or MMR proficient SH 1837/1 (with *chc* wild type), on automated fluorescent DNA sequencers using a labeled M13 primer and SSR: **(A)** Sti 046 or **(B)** Sti 054; the bands indicating SSR instability (MSI) are indicated by red arrows and parental, control and respective MMR-deficient genotypes are grouped in red rectangles (for SSR markers and their location on potato chromosomes, see Supplementary Table S5).

Molecular analysis using 96 SSR markers (12 with an unknown chromosome location) generally revealed little polymorphism, with only 11 having a polymorphic profile. Only six SSR markers were unstable (Supplementary Table S5). The polymorphic SSR markers were used to evaluate hybridity of regenerated plants. The SSR markers Sti046 and Sti054 were selected to exemplify MSI in the SHs with MMR deficient *chc* (Supplementary Table S5). The ‘mutator’ phenotypes of SHs with MMR deficiency (DkDN: 5.3, 5.11, 5.17, 5.25, 11.24, 11.26, and 11.34, DeDN: 5.5, 11.5 or DkAS: 10.5 and 10.8), are shown alongside that of their respective parents in Figure 6A. Similar SHs (with few exceptions) and their parents analyzed using Sti054 are presented in Figure 6B. As an additional negative control to that of the parents, SH 1837/1 between potato cv. ‘Delikat’ and wild type *chc* was used to confirm the stability of SSR profiles. One MMR deficient SH, DkDN 5.6, shows for both SSR markers, profiles similar to those of its parent ‘Delikat,’ but the correlation between plant height and ploidy level indicates it has an abnormal phenotype, as the plants are very big and have large leaves. This gigantic growth is not correlated with ploidy level, as this SH is nearly tetraploid but not considered to be a ‘mutator’ phenotype (Figures 5, 6, Table 2, and Supplementary Figure S3).

#### Evaluation of *MSH2* Activity Using RT-qPCR

**FIGURE 7:**
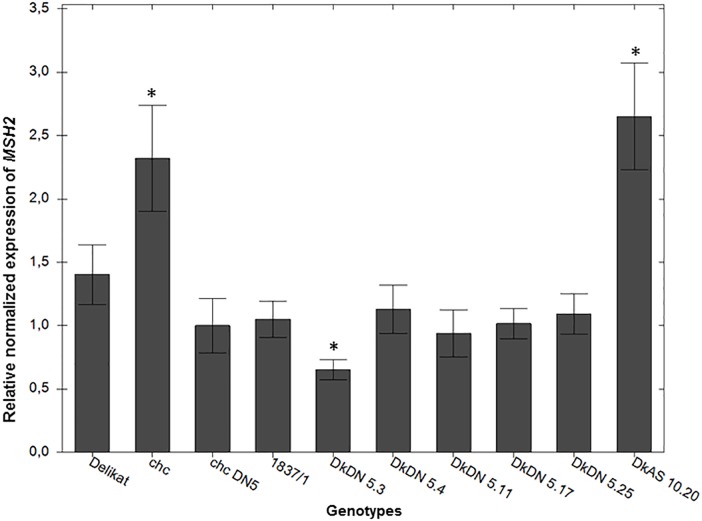
Relative normalized expression of the *MSH2* gene in potato somatic hybrids with and without MMR deficiency in comparison with high leptine producer (HL) *Solanum chacoense* (*chc*) with a mutation in the *MSH2* gene (MMR deficient) DN 5 and the wild type *chc* and *S. tuberosum* cv. ‘Delikat.’ Bars are the standard error of the mean of the replicates (±SEM) ^∗^ significant at *p* < 0.05 when compared to transgenic *chc* DN 5.

The activity of the *MSH*2 gene in SHs was reduced when the DN mutation of the *AtMSH*2 gene was transformed in the *chc* parent. The relative expression of the *MSH2* gene, differed in the SHs (Figure 7). Compared to the MMR deficient *chc* DN 5, used as a control, the relative expression of the *MSH2* gene was significantly decreased in the MMR deficient somatic hybrid DKDN 5.3, in which the relative expression was 0.64 (*p* = 0.001). This SH showed the strongest reduction in *MSH2* expression. The expression of *MSH2* in wild type *chc* was greater (2.29) than in transgenic *chc* DN 5 (*p* = 0.0001), which demonstrates that the dominant negative mutation in the *AtMSH*2 gene reduces its activity. When, the relative expression is compared with that in the wild parental plants (*S. tuberosum* and *S. chacoense* HL), the expression of *MSH2* is significantly downregulated in DN mutant SHs. As for the extreme genotype with a dwarf phenotype, DkAS 10.20, the *MSH2* activity is greatly increased, an alteration which might be associated with the mixoploidy of this particular hybrid (Figure 7).

## Discussion

DNA MMR is involved in maintaining DNA integrity in all organisms and its proteins have been highly conserved during evolution (Surtees et al., 2004). A defective MMR is the cause of Lynch Syndrome in humans, which makes them more likely to develop certain types of cancer (Nicolaides et al., 1998; Modrich, 2006; Narine et al., 2007; Seifert et al., 2008). Plants being sessile, have to cope with external and endogenous mutagens that cause replication errors (chemicals such as heavy metals, alkylating agents or reactive oxygen species, ROS and solar UV-B radiation etc.). Moreover, plants unlike animals do not have a separate germline and consequently mutations arising during vegetative growth might be transmitted to the next generation as reported in maize (Das et al., 1990). In order to maintain DNA stability plants need to be very efficient at repairing DNA (Britt, 1999). The post-replication repair of DNA involves MMR proteins forming mismatch recognition heterodimers, of which one constant element is MSH2 (Leonard et al., 2003). In this study the *MSH*2 gene isolated from *Arabidopsis thaliana* was transformed into the wild species *chc*. For the induction of MMR deficiency *Agrobacterium*-mediated transformation was used (Rakosy-Tican et al., 2004) (Figure 1A). There are many accessions of the species *chc*, but in order to achieve our final goal it was important to use the high leptine producing (HL) GenBank accession PI 458310. The specific glycoalkaloids, leptines, synthetized only in green tissues of plants are known to be toxic and to deter CPB (Sinden et al., 1986; Molnár et al., 2016). Obtaining cultivars of potato that are resistant to CPB is important because this pest is extremely adaptable and voracious and has developed resistance to 53 insecticides worldwide (Alyokhin et al., 2008).

### Induction and Identification of Mutant Phenotypes in Somatic Hybrids With MMR Deficiency

Mismatch repair deficiency is thought to induce mutagenesis and increase homeologous recombination in hybrids between closely related species (Rayssiguier et al., 1989; Matic et al., 1994; Spampinato et al., 2009; Dong et al., 2013). It is proposed that MMR deficiency increases plant diversity a feature that is important for plant breeding (Chao et al., 2005; Wijnker and de Jong, 2008). In our research on a very important crop, potato, the production of large numbers of SHs with MMR deficient *chc* and the analysis of their phenotypes *in vitro* and *ex vitro* in comparison with those of wild type SHs (controls) and their parents indicate that a large number and diversity of mutations, i.e., ‘mutator’ phenotypes, are induced. Only those mutations with MSI, the molecular signature of MMR deficiency, were considered to be ‘mutator’ phenotypes as the other phenotypic abnormalities are caused by somatic hybridization and its complex genetic interactions (Harms, 1983). MMR deficiency coupled with somaclonal variation, induced during long term culture *in vitro* and maintenance as micro-tubers could also cause the SH clones to vary, but our data on ploidy level indicates that SHs remain stable for at least 7 years. If all the viable SHs grown on in a greenhouse are considered (Table 1), 40% showed at least one phenotypic mutation (10 SHs highlighted in red in Table 2), but double or even triple changes in phenotype were also recorded. In addition, a callus like shoot was regenerated *in vitro* from a MMR deficient *chc*, and a chimeric albino was recorded for one SH with an AS MMR deficiency. Similar mutations are reported in *Arabidopsis thaliana* when the *MSH*2 gene mutates (Hoffman et al., 2004) or is silenced (Leonard et al., 2003). Artificial miRNA based on comparative sequences of *MSH*2 genes from *Solanaceae* transformed into two tobacco species are not as effective in inducing a ‘mutator’ phenotype (Van Marcke and Angenon, 2013). Recent data on whole genome sequencing indicates that MMR deficient *A. thaliana* is more prone to the single nucleotide variants in genes mutating and that the intergenic regions are less affected (Belfield et al., 2018). This supports our results and accounts for the occurrence of a large number of mutant phenotypes in first generation fusion hybrids, when the MMR deficiency in one parent was induced by an AS or DN mutation of *MSH*2. The above mentioned results open new avenues in the study of cancer, plant evolution and crop breeding.

In this study, it was not easy to evaluate lethal mutations as somatic hybridization can generate somatic incompatibility and a great diversity in the ability of each SH clone to regenerate (Rakosy-Tican et al., 2015). Nevertheless, since some aberrant phenotypes are also caused by somatic incompatibility in fusion hybrids (Harms, 1983), lack of a correlation between plant height and ploidy level was also used to detect the SHs with ‘mutator’ phenotypes. Another issue raised by the genetic transformation is the site of the integration of transgenic DNA, however, was not assessed in this study. There was, however, a possibility that mutations caused by transgenesis would be detectable in the selected transgenic *chc* parent. The phenotypes of three transgenic *chc* clones used in electrofusion were not more variable than those of the wild species. For a few SHs only MSI and lack of flower development supported the notion that this was due to mutations caused by the MMR deficient *chc* parent (Table 2). The combined effect of somatic incompatibility and MMR deficiency results in the first generation fusion hybrids being very variable. Moreover the presence of the *npt*II gene, based on its specific phenotype, root development on media containing kanamycin, proved that with two exceptions, all clones considered as ‘mutator’ phenotypes and positive for MSI are also able to develop roots in the presence of kanamycin. The two exceptions, which were positive for MSI might have lost the transgenes during somatic fusion or later during cell proliferation or regeneration, but this hypothesis needs further investigation. The analysis of the activity of the *MSH2* gene using RT-qPCR confirms that its activity in a DN transgenic clone of *chc* was reduced compared with that in the wild type parent, and a reduction in activity in SHs with MMR deficiency compared to both potato and *chc* wild type parents. This data indicates that the DN mutation of the *AtMSH2* gene can reduce the general activity of normal alleles of *MSH2* in somatic hybrids. Although the RT-qPCR analysis revealed an increased level of the *MSH2* transcript in the dwarf phenotype of SH, DkAS 10.20, other AS hybrids did not show a reduction in gene activity. For this particular hybrid its mixoploidy and resultant genetic instability might account for the increase in the levels of the *MSH2* transcript and possibly of MSI, as occurs in rice hybrids with intergenic introgressions (Dong et al., 2013). Many of the SHs were aneuploid, thus they might be expected to have high mutation rates as is the case in human cancers (Knutsen et al., 2010). In knockout mice a defective MMR is compatible with normal growth and development (Nicolaides et al., 1998). The majority of the shoots of potato SHs grew and developed normally, but some did not flower or exhibited stunted growth. Nevertheless, a dominant negative mutant allele can destabilize MMR even in the presence of a normal allele (Nicolaides et al., 1998), as seems to be the case in our SHs in which four normal alleles from the potato tetraploid parent were out competed or inactivated by the DN *AtMSH*2 gene from the transgenic *chc* diploid parent. Moreover, the phenotypes of the wild type somatic hybrids between two species of the same or different *chc* accessions previously produced in our laboratory are not similar to those described here as ‘mutator’ phenotypes. Previous data also indicate that the *chc* parent is unable to regenerate plants on the media used for fusion product regeneration (Rakosy-Tican and Aurori, 2015).

The flow cytometry analysis of ploidy levels revealed MMR deficient and MMR proficient SHs. The genome constitution of the majority of the shoots of fusion hybrids that included *chc* with MMR deficiency were tetraploid, or near tetraploid, whereas those of the wild type somatic hybrids were as expected for 4x potato + 2x *chc* fusions, mainly hexaploid or near hexaploid. Although, SHs with MMR deficiency seem to loose chromosomes more than wild type SHs, this refutes the previous assumption that recombination-dependent chromosome loss is stimulated by a proficient MMR system (Chambers et al., 1996). The loss of chromosomes and introgressions of wild species genes into the recipient potato are under investigation using GISH during mitosis and meiosis. The chromosome counts confirmed that the majority of the tetraploid SHs are in fact aneuploids. Mixoploidy was also quite frequent recorded among SHs, with or without MMR deficiency, and this genomic status can also cause genetic instability as was the case of SHs without MSI derived from wild type *chc*, i.e., DeC 7 (Table 2). RT-qPCR analysis of another wild type SH 1837/1 revealed it had a reduced *MSH2* transcript level compared to wild type *chc*, but the level was not significantly different from that of the wild type potato parent ‘Delikat’ (Figure 7). Another SH DkDN 5.6 has the same SSR profile as cv. Delikat, but its height is not correlated with its ploidy level and might be the result of introgressions from the wild parent (an heterosis-like effect). This needs further investigation at cytogenetic and molecular levels. A similar case is described for hybrids of rice, where introgressions from a wild species alters the MMR activity (Dong et al., 2013). The combined effects of somatic fusion, aneuploidy/mixoploidy and a deficient MMR system is more likely to account for the abnormal phenotypes that developed in a greenhouse in this study, a total of 17 SHs out of 25, of which only ten exhibited MSI and eight resistance to kanamycin (Table 2). When plant height was correlated with plant ploidy level 12 SHs grew in an unexpected way (Figure 5). Quite a diverse array of somatic phenotypic mutations was identified among the SHs that were grown in a greenhouse: dwarf plants, bushiness, abnormal leaves (small oval leaflets, large, conjoint or curled leaves), gigantic plants, variable deep purple or small deformed tubers and a different flower development. The great variation in phenotypes, with both stunted and gargantuan growth and increase in yield might be useful traits for breeding. Similar phenotypes are described in *Arabidopsis* when mutated MSH1 causes alterations in the epigenome and heritable changes in plant growth (Virdi et al., 2015).

### The Role of MMR Deficiency in Increasing Homeologous Recombination

**FIGURE 8:**
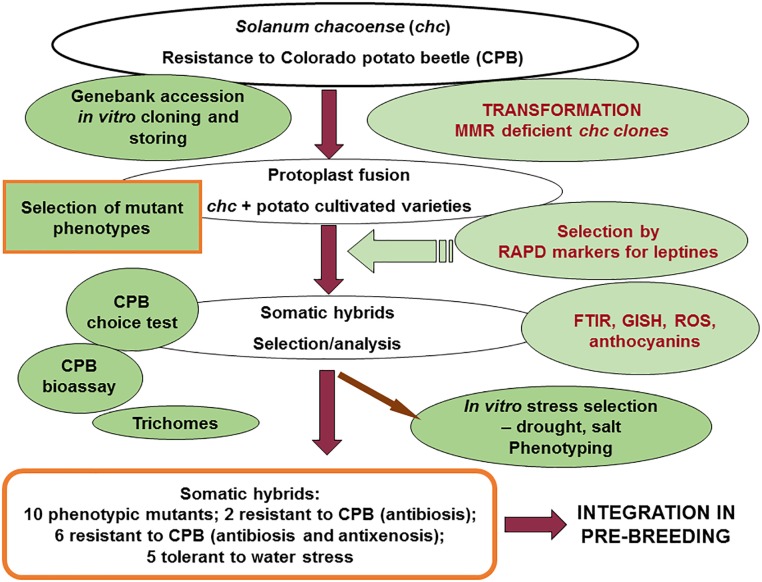
Schematic representation of the combinatorial biotechnology program and its results for somatic hybrids between potato and high leptine producing *S. chacoense*. The data for mutant phenotypes have red borders; the genotypes that were produced as a result of this complex scheme are underlined in red (see also Table 2 for both CPB resistant and drought tolerant genotypes shown in bold).

In various organisms, it is known that MMR is involved in reducing recombination between homeologous sequences, which is known as antirecombination (Harfe and Jinks-Robertson, 2000; Schoffield and Hsieh, 2003). In this study, this capacity of MMR deficient clones of *chc* in the somatic hybridization experiments with commercial cultivars of potato, was used to increase homeologous recombination between two related genomes. The final goal was to use homeologous recombination to increase the introgression of the genes responsible for the biosynthesis of leptines or other resistance genes and so produce pre-breeding lines resistant to CPB and/or other diseases or pests. The analysis of the level of antibiosis and antixenosis against CPB supports an increase in the introgression of these traits in SHs with MMR deficiency (Molnár et al., 2016; data in Table 2). These data confirm an increase of introgressions in the genomes of two related species, which augments the value of this unique plant material. Similar results are reported for sexual crosses and embryo rescue in tomato when the wild species *S. lycopersicoides* is used (Tam et al., 2011). Moreover, the data on tolerance of drought after selection *in vitro* using media with two concentrations of PEG and then *ex vitro* cultivation in a greenhouse using a phenotyping platform, indicates that although none of the parents were tolerant a few of the MMR deficient potato SHs exhibited tolerance and were able to recover after exposure to drought (Table 2; detailed data not shown). Under drought conditions the best performing SH clone, DkAS 10.13, did not produce any other mutant phenotype. In general these results support our previous proposed strategy of combinatorial biotechnology as a means of transferring multiple resistance traits from a wild species reservoir into the gene pool of a crop (Rakosy-Tican et al., 2016) (Figure 8). In the combinatorial biotechnology scheme shown in Figure 8 the most important steps are the selection of wild species accessions resistant to CPB, plants resistant *in vitro*, cloning and storing. Then only resistant clones are used for genetic transformation to induce MMR deficiency or for protoplast fusion. Subsequently, fusion is performed with varieties of cultivated potato in order to insert a resistance trait into the background of a cultivar of potato (cv. ‘Delikat’ or ‘Désirée’). The selection of somatic hybrid plants was done using the RAPD markers described earlier as markers for the biosynthesis of leptines (OPT-20), and other techniques like Fourier Transform Infrared Spectroscopy (FTIR), genome *in situ* hybridization (GISH) during mitosis and meiosis (under way), analysis of different types of trichomes and their density and biochemical characterization: anthocyanins, reactive oxygen species (ROS), etc. (Rakosy-Tican et al., 2016). The second most important to ‘mutator’ phenotype screenings was the analysis of antixenosis in choice tests using adult CPB and antibiosis using a laboratory bioassay in which the development of larvae, pupae and adult CPB and their fertility was recorded (Molnár et al., 2016). In order to select genotypes that are tolerant of drought, both *in vitro* selection and greenhouse phenotyping were used. For salt tolerance, only *in vitro* selection was used. Further analysis of meiosis, genetic introgressions and resistance to other biotic and/or abiotic stresses will guarantee development of both basic as well as applied results (see scheme detailed in Figure 8).

The strategy described here might be successfully applied to other species of plants for which somatic hybridization can be used to induce novel output traits for agriculture and bring new phenotypic data on MMR deficiency effects in artificially merged protoplasts and their derived regenerated colonies, calluses and plants.

## Conclusion

In this study we demonstrate that: 1) new potato phenotypes can be co-induced by somatic hybridization and MMR-deficiency using transgenic *S. chacoense* carrying the *AtMSH2* gene in antisense orientation or as a dominant negative mutant (68% of the analyzed SHs); 2) ten ‘mutator’ phenotypes caused by uniparental MMR-deficiency were identified by using different molecular (microsatellite instability, *MSH2* gene activity), cytogenetic (correlation between ploidy and plant high) and *in vitro* assays (root growth on kanamycin media) – they represent 40% of the analyzed SHs with MMR deficiency; together MMR deficiency and somatic hybridization caused 68% phenotype abnormalities; in contrast, only 12.5% of control MMR proficient SHs show phenotype abnormalities i.e. one plant out of eight wild type SHs 3) MMR-deficiency increases introgression of useful traits like CPB resistance and tolerance of drought in the resulted somatic hybrids; mutants with a practical value were selected and will be integrated into a large scheme for producing pre-breeding multiple resistant potato.

## Author Contributions

ER-T designed the experiments and wrote the manuscript. AA performed the electrofusion, regeneration and evaluation, and chose the fusion partners and carried out the experiments on the growth of the colonies. IM performed the ploidy analysis using chromosomes counts, flow cytometry, and related statistics. EL-B contributed to the assessments in the greenhouse and kanamycin tests. EL-B and OA contributed to the MSI analysis. EL-B, FH, and TS contributed to the RT-qPCR with related statistics. RT contributed to the first flow cytometry and molecular analysis. GA participated in the development of DNA constructs and molecular analysis of transgenic clones. IF contributed the initial idea and constructs. All authors checked the manuscript and approved it and contributed to the revision of the data.

## Conflict of Interest Statement

The authors declare that the research was conducted in the absence of any commercial or financial relationships that could be construed as a potential conflict of interest.

## Footnotes

1 https://www.ncbi.nlm.nih.gov/tools/primer-blast/
