# Supplementary material for: New Phenotypes of Potato Co-induced by Mismatch Repair Deficiency and Somatic Hybridization
Source: Front Plant Sci. 2019 Jan 22;10:3. doi: 10.3389/fpls.2019.00003 (PMC6349821; doi:10.3389/fpls.2019.00003)
Supplement: Supplementary file 1 [file Table_1.pdf]

Supplementary Table S1 Evaluation of transgenic *Solanum chacoense* PI 458310 clones by culture on 50 mg L^{-1} kanamycin (kan) containing media or by RT-PCR; development of root growth and callus was assessed after one month in culture; ND = not determined

**Response**	**The contruct with dominant negative *Atmsh*2 mutant gene (DN)**	**The construct with antisense *Atmsh*2 gene (AS)**
**Total number of plants**	14	15
**Plants rooted in absence of kan**	12	6
**Plants rooted with kan**	2	9
**Development of callus on kan media**	ND	5
***Msh*2 RT-PCR product +**	3	5
**Trangenic clones selected for fusion experiments**	2<br>(DN 5 and DN 11)	1<br>(AS 10)
