# Supplementary material for: New Phenotypes of Potato Co-induced by Mismatch Repair Deficiency and Somatic Hybridization
Source: Front Plant Sci. 2019 Jan 22;10:3. doi: 10.3389/fpls.2019.00003 (PMC6349821; doi:10.3389/fpls.2019.00003)
Supplement: Supplementary file 2 [file Table_2.pdf]

Supplementary Table S2 Total number of somatic hybrids selected on the base of vigorous growth in the fusion combinations involving potato cv. 'Delikat' (Dk) and wild type (WT) or MMR defficient *Solanum chacoense* PI 458310 (c*hc*) with *Atmsh*2 antisense (AS) or dominant negative (DN) gene; * plating efficiency = number of cell colonies regenerated per 100 plated protoplasts after two weeks in culture

**Fusion partners**	**Protoplast density pp/ml**	**Plating efficiency (%)***	**Total number of plants (n)**
Dk + *chc* WT	$6.2x10^4$	21.7	13
Dk + *chc* DN	$6x10^4$	10.8	67
Dk + *chc* AS	$6.2x10^4$	5.6	57
