# Supplementary material for: New Phenotypes of Potato Co-induced by Mismatch Repair Deficiency and Somatic Hybridization
Source: Front Plant Sci. 2019 Jan 22;10:3. doi: 10.3389/fpls.2019.00003 (PMC6349821; doi:10.3389/fpls.2019.00003)
Supplement: Supplementary file 3 [file Table_3.pdf]

Supplementary Table S3 Total somatic hybrid plants regenerated, when all shoots were harvested that arose from calluses, for different fusion combinations with or without MMR deficient *Solanum chacoense* HL (*chc*); Total mutation frequency (%) was calculated, based on total number of regenerated plants, after analyzing the plants *in vitro* and *ex vitro*. Dk – *S. tuberosum* cv. 'Delikat'; De – cv. 'Désirée'. Somatic hybrids with *chc*: AS – clones carrying *AtMSH*2 antisense gene; DN – clones carrying dominant negative *AtMSH*2 gene

**Somatic hybrids (combination)**	**Code of the hybrid clones**	**Total number of plants (n)**	**Mutation frequency (%)**
**Dk + *chc* wild type**	DkC	9	0*
**Dk + *chc* AS 10**	DkAS 10	91	5.5
**Dk + *chc* DN 5**	DkDN 5	42	11.9
**Dk + *chc* DN 11**	DkDN 11	38	10.5
**De + *chc* wild type**	DeC	10	0*
**De + *chc* AS 10**	DeAS 10	0	0
**De + *chc* DN 5**	DeDN 5	15	6.6
**De + *chc* DN 11**	DeDN 11	34	2.9

*Changes in phenotype observed in DkC or DeC control somatic hybrids, involving wild type *Solanum chacoense*, were considered a result of somatic incompatibility and were not included in the evaluation; n = total number of regenerated clones, some of which did not survived micropropagation and storing
