# Supplementary material for: New Phenotypes of Potato Co-induced by Mismatch Repair Deficiency and Somatic Hybridization
Source: Front Plant Sci. 2019 Jan 22;10:3. doi: 10.3389/fpls.2019.00003 (PMC6349821; doi:10.3389/fpls.2019.00003)
Supplement: Supplementary file 4 [file Table_4.pdf]

Supplementary Table S4 The ploidy of the MMR deficient somatic hybrids and parental wild type and transgenic clones assayed by flow cytometry, as means of at least two measurements; some somatic hybrids were reassessed after 7 years of *in vitro* culture and storage as micro - tubers; AS = antisense and DN = dominant negative *Atmsh2* gene

Somatic hybrids or parents	Clone	Ploidy<br>Initial (after 7years)
***Solanum tuberosum* 'Delikat'**	Parent	4x (4x)
***Solanum tuberosum* 'Désirée'**	Parent	4x (4x)
***Solanum chacoense* HL**	Parent	2x (2x)
***Solanum chacoense* HL AS 10**	Transgenic clone	4x
***Solanum chacoense* HL DN 5**	Transgenic clone	2x (2x)
***Solanum chacoense* HL DN 11**	Transgenic clone	2x (2x)
**'Delikat'+ *S. chacoense* HL AS 10**	DkAS 10.1	4x -mixo
	DkAS 10.2	4x
	DkAS 10.5	4x (4x)
	DkAS 10.6	4x
	DkAS 10.7	4x-6x mixo
	DkAS 10.8	4x (4x)
	DkAS 10.9	6x-8x mixo
	DkAS 10.10	4x
	DkAS 10.11	4x mixo (4x)
	DkAS 10.12	4x
	DkAS 10.13	4x
	DkAS 10.17	4x
	DkAS 10.18	5x-6x mixo
	DkAS 10.35	4x-5x mixo
	DkAS 10.40	4x
	DkAS 10.51	4x
**'Delikat' + *S. chacoense* HL DN 5**	DkDN 5.2	6x
	DkDN 5.3	6x (mixo)
	DkDN 5.5	4x-6x mixo
	DkDN 5.6	Nd (4x)
	DkDN 5 7	4x-6x mixo
	DkDN 5.8	4x
	DkDN 5.9	6x-8x mixo
	DkDN 5.10	2x
	DkDN 5.11	6x (6x)
	DkDN 5.12	4x
	DkDN 5.16	4x
	DkDN 5.17	6x
	DkDN 5.18	4x
	DkDN 5.23	4x
	DkDN 5.24	4x-6x mixo
	DkDN 5.25	6x-8x mixo (4x-6x mixo)
	DkDN 5.26	4x
	DkDN 5.27	6x

**'Delikat' + *S. chacoense* HL DN 11**	DkDN 11.1	4x-5x mixo
	DkDN 11.3	6x
	DkDN 11.10	4x-6x mixo
	DkDN 11.11	4x
	DkDN 11.17	2x
	DkDN 11.18	4x
	DkDN 11.22	4x
	DkDN 11.23	4x-6x mixo
	DkDN 11.24	Nd (6x)
	DkDN 11.26	5x-6x mixo
	DkDN 11.27	5x-6x mixo
	DkDN 11.30	6x
	DkDN 11.31	4x
	DkDN 11.33	4x
	DkDN 11.34	4x (4-5x mixo)
	DkDN 11.35	4x
	DkDN 11.39	5x-6x mixo
**'Delikat' + *Solanum chacoense* HL**	DkC 5	4x
**'Désirée' + *S. chacoense* HL DN 5**	DeDN 5.1	4x
	DeDN 5.3	5x-6x mixo
	DeDN 5.5	4x
**'Désirée' + *S. chacoense* HL DN 11**	DeDN 11.1	4x
	DeDN 11.2	6x
	DeDN 11.5	Nd (4x)
	DeDN 11.6	2x
	DeDN 11.11	5x-6x mixo
	DeDN 11.12	5x-6x mixo
	DeDN 11.15	6x
	DeDN 11.17	4x
	DeDN 11.19	4x
	DeDN 11.22	4x
	DeDN 11.23	4x
	DeDN 11.25	2x
	DeDN 11.28	4x
**'Désirée' + *Solanum chacoense* HL**	DeC 2	5x, 6x (mixo) (mixo)
	DeC 3	5x-6x mixo
	DeC 4	6x
	DeC 5	4x-5x mixo
	DeC 6	5x-6x mixo
	DeC 7	4x-5x mixo (mixo)
	DeC 8	5x-6x mixo
	DeC 9	5x
	DeC 11	6x
	DeC 12	5x
	DeC 13	5x-6x mixo
