# Supplementary material for: New Phenotypes of Potato Co-induced by Mismatch Repair Deficiency and Somatic Hybridization
Source: Front Plant Sci. 2019 Jan 22;10:3. doi: 10.3389/fpls.2019.00003 (PMC6349821; doi:10.3389/fpls.2019.00003)
Supplement: Supplementary file 5 [file Table_5.pdf]

Supplementary Table S5 SSR markers used to analyze the somatic hybrids between wild type or MMR deficient *Solanum chacoense* HL and potato, indicating their position on potato chromosomes; the markers which show instability are highlighted in yellow while the ones showing polymorphism are in green; ND – chromosome position not determined; [a] – Suspected multilocus marker, additional location possible

Chromosome	SSR name	Repeat	References
1	StI009	$(AGC)_n$ $(AAC)_n$	Feingold *et al.*, 2005
	StI031	$(TCA)_n$	
	StI043	$(AAC)_{imp}$	
	StI062	$(GAA)_n$	
	STM0015	$(AC)_{13}$ $(AT)_{10}$	Milbourne *et al.*, 1998
	STM1029	$(C)_{12}$	
	STM1049	$(ATA)_6$	
	STM2020	$(TAA)_6$	
	STM2030	$(CA)_3$ $(TA)_5$	
	LeatpacA	$(TA)_7$	
	STM5127	$(TCT)_n$	Ghislain *et al.*, 2009
	STG0016	$(AGA)_8$	
	STM5136	$(AGA)_5$	Reid and Kerr, 2007
2	StI024	$(CAA)_n$	Feingold *et al.*, 2005
	StI029	$(CA)_{imp}$ $(TC)_{imp}$	
	StI052	$(CTT)_n$	
	StI053	$(AT)_{imp}$	
	STM0038	$(TA)_4$ $(TG)_{12}$	Milbourne *et al.*, 1998
	STM2022	$(CAA)_3$…$(CAA)_3$	
	STM3001	$(TC)_5$	
	STM1064	$(TA)_n$ $(TG)_n$ GT $(TG)_n$	Ghislain *et al.*, 2009
	STM5114	$(ACC)_n$	
3	StI013	$(ACC)_n$	Feingold *et al.*, 2005
	StI050	$(ATA)_n$	
	StI060	$(ATA)_n$	
	StI061	$(GAA)_n$	
	STM0040	(AT) 20AG…$(AT)_2$ $(GT)_{11}$	Milbourne *et al.*, 1998
	StI001	$(AAT)_n$	Feingold *et al.*, 2005
	StI012	$(ATT)_n$	

**4**	StI026	$(TA)_n$ $(GT)_n$	
	StI055	$(AAG)_n$	
**5**	StI006	$(AT)_n$ $(GT)_n$	
	StI032	$(GGA)_n$	
	StI049	$(TCTT)_n$	
	STM0013	$(AC)_{23}$ $(AT)_6$ $(AG)_9$	Milbourne *et al.*, 1998
**6**	StI004	$(AAG)_n$	Feingold *et al.*, 2005
	StI011	$(AC)_n$ $(AT)_n$	
	StI015	$(AT)_n(AG)_n(AT)_n$	
	StI016	$(CCT)_n$	
	StI021	$(CAT)_n$	
	StI045	$(CCT)_n$	
	StI059	$(AT)_n$	
	STM0025	$(TG)_{13}$	Milbourne *et al.*, 1998
	STM0001	$(TG)_4$ $(TC)_2$ $(TG)_5$	
**7**	StI008	$(AGC)_n$	Feingold *et al.*, 2005
	StI025	$(CTCC)_n$	
	StI033	$(AGG)_n$	
	StI040	$(ACA)_n$ $(GCA)_n$	
	StI064	$(AT)_n$ $(GT)_n$	
	STM0028	$(AC)_{12}$ $(AT)_5$ $(AG)_8$	Milbourne *et al.*, 1998
	STM0031	$(AC)_5$…$AC)_3$ (GCAC). $(AC)_2$ $(GCAC)_2$	
**7,12**	STM0014	$(GT)_5$ $(AT)_7$ $(GT)_{10}$	
**8**	StI003	$(ACC)_n$	Feingold *et al.*, 2005
	StI022	$(ACCCG)_n$	
	StI0027	$(GAA)_n$	
	StI047	$(TAA)_n$	
	StI048	$(GAT)_n$	
	STM0024	$(GTT)_5$…$(AC)_{12}$ $(AT)_6$	Milbourne *et al.*, 1998
	STM1001	$(A)_{19}$	
	STM1016	$(TCT)_9$	
	STM1024	$(TTG)_6$	
	STM1104	$(TCT)_5$	

**9**	StI002	(ATT)n	Feingold *et al.*, 2005
	StI014	$(TGG)_n$ $(AGG)_n$	
	StI057	$(AGG)_n$	
	STM0010	$(TG)_7$ $(TA)_6$ $(TG)_4TA$ $(TG)_5$	Milbourne *et al.*, 1998
	STM1017	$(ATT)_5$	
**10**	StI023	$(GGC)_n$ $(GGT)_n$	Feingold *et al.*, 2005
	STG0025	$(AAAC)_n$	Ghislain *et al.*, 2009
	STM0051	$(AC)_7$…$(AC)_7$ $(AT)_4$	Milbourne *et al.*, 1998
	STM1106	$(ATT)_{13}$	
**11**	StI017	$(CAT)_n$ $(TAG)_n$ $(AAG)_n$	Feingold *et al.*, 2005
	StI028	$(CAA)_{imp}$	
	StI039	$(AAC)_n$ $(AAT)_n$	
	StI041	$(GAA)_n$	
**11**[a]	StI046	$(GAT)_n$	
**11**	STG0001	$(CT)_n$	Ghislain *et al.*, 2009
	STM0037	$(TC)_5$ $(AC)_6AA$…$(AC)_7$ $(AT)_4$	Milbourne *et al.*, 1998
**12**	StI007	$(GTT)_n$ $(GAT)_n$	Feingold *et al.*, 2005
	StI030	$(ATT)_n$	
	StI054	$(CCA)_n$	
	StI063	$(GAT)_{imp}$	
	STM0007	$(AC)_9$	Milbourne *et al.*, 1998
	STM0032	$(AC)_7$…$(AC)_5$	
**ND**	StI035	$(TTC)_n$	Feingold *et al.*, 2005
	StI042	$(AT)_n$	
	STM0002	$(TA)_8$ $(TG)_8$ TC $(TG)_7$	Milbourne *et al.*, 1998
	STM0006	$(AC)_{14}$ $(AT)_5$	
	STM0011	$(TG)_7$ $(TA)_6$	
	STM0020	$(AC)_{10}$	
	STM0021	$(GT)_{11}$ $(G)_{14}$	
	STM0023	$(TG)_3$ $(TA)_6$ $(TG)_{12}$	
	STM0045	$(A)_8$ $(TA)_2$ $(TG)_{10}$	
	STM0046	$(GC)_4$ $(AC)_{17}$ $(AT)_5$	
	STM0047	$(AC)_{13}$ $(AT)_9$	
	STM0050	$(AC)_{18}$	

Exemplified in Fig. 6:

*StI046 marker with the primers:

F: CAGAGGATGCTGATGGACCT and R: GGAGCAGTTGAGGGCTTCTT

** StI054 marker with the primers:

F: GCCACTATGCAAGCCCATTTG and R: GGGTCGATGTTTCGGTTGAG
