# Supplementary material for: New Phenotypes of Potato Co-induced by Mismatch Repair Deficiency and Somatic Hybridization
Source: Front Plant Sci. 2019 Jan 22;10:3. doi: 10.3389/fpls.2019.00003 (PMC6349821; doi:10.3389/fpls.2019.00003)
Supplement: Supplementary file 6 [file Image_1.pdf]

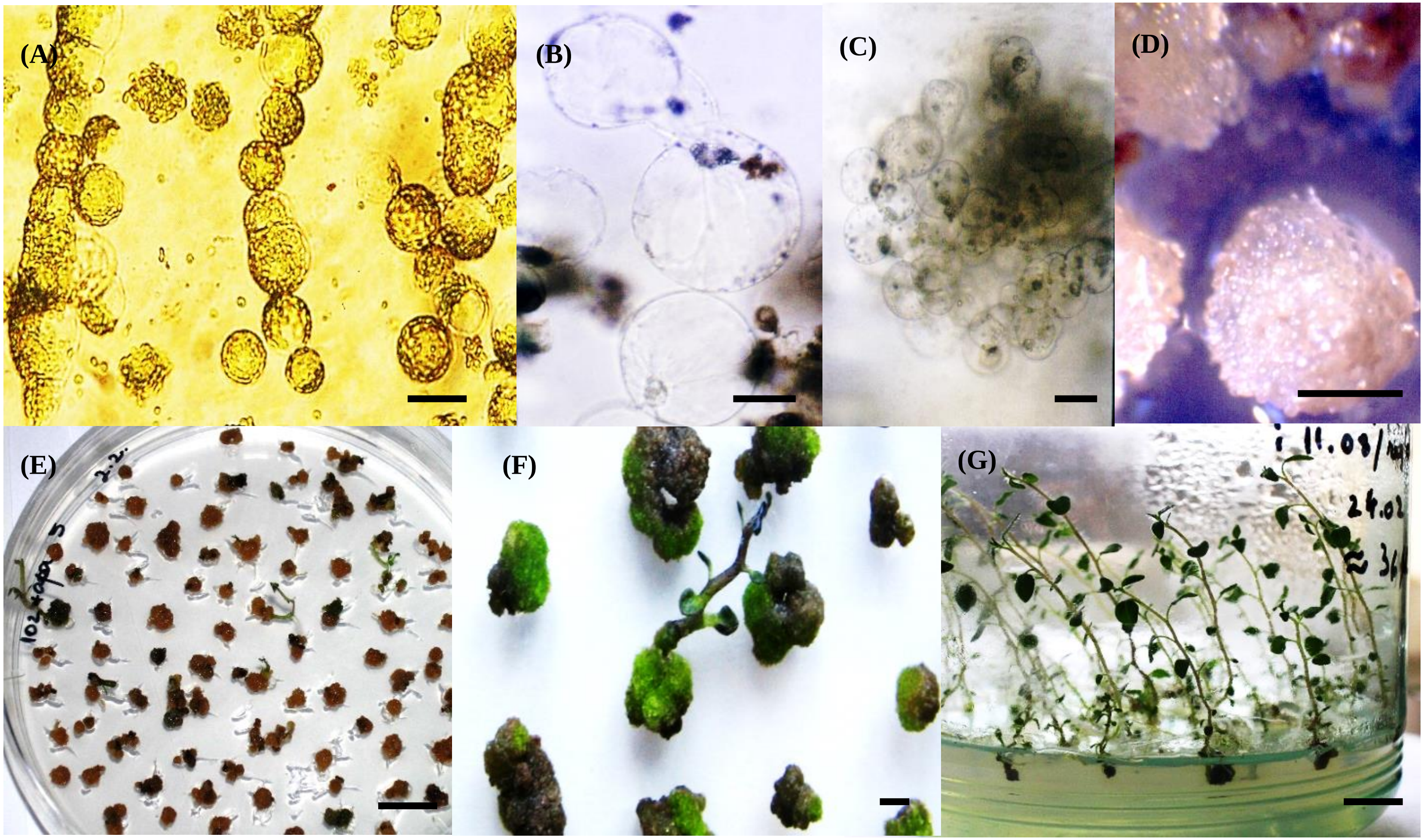

Supplementary Fig. S1 Electrofusion of mesophyll protoplasts **(A)** of the two parental species potato + S. *chacoense* and different developmental stages to plant regeneration: **(B)** cell division; **(C)** cell colonies; **(D)** and **(E)** - micro- and macro-calluses; **(F)** detail of callus with first arising shoot and **(G)** regenerated shoots; bars = 100 μm (A,B and C), 1 mm (D), 1 cm (E,F and G)
