# Supplementary material for: New Phenotypes of Potato Co-induced by Mismatch Repair Deficiency and Somatic Hybridization
Source: Front Plant Sci. 2019 Jan 22;10:3. doi: 10.3389/fpls.2019.00003 (PMC6349821; doi:10.3389/fpls.2019.00003)
Supplement: Supplementary file 7 [file Image_2.pdf]

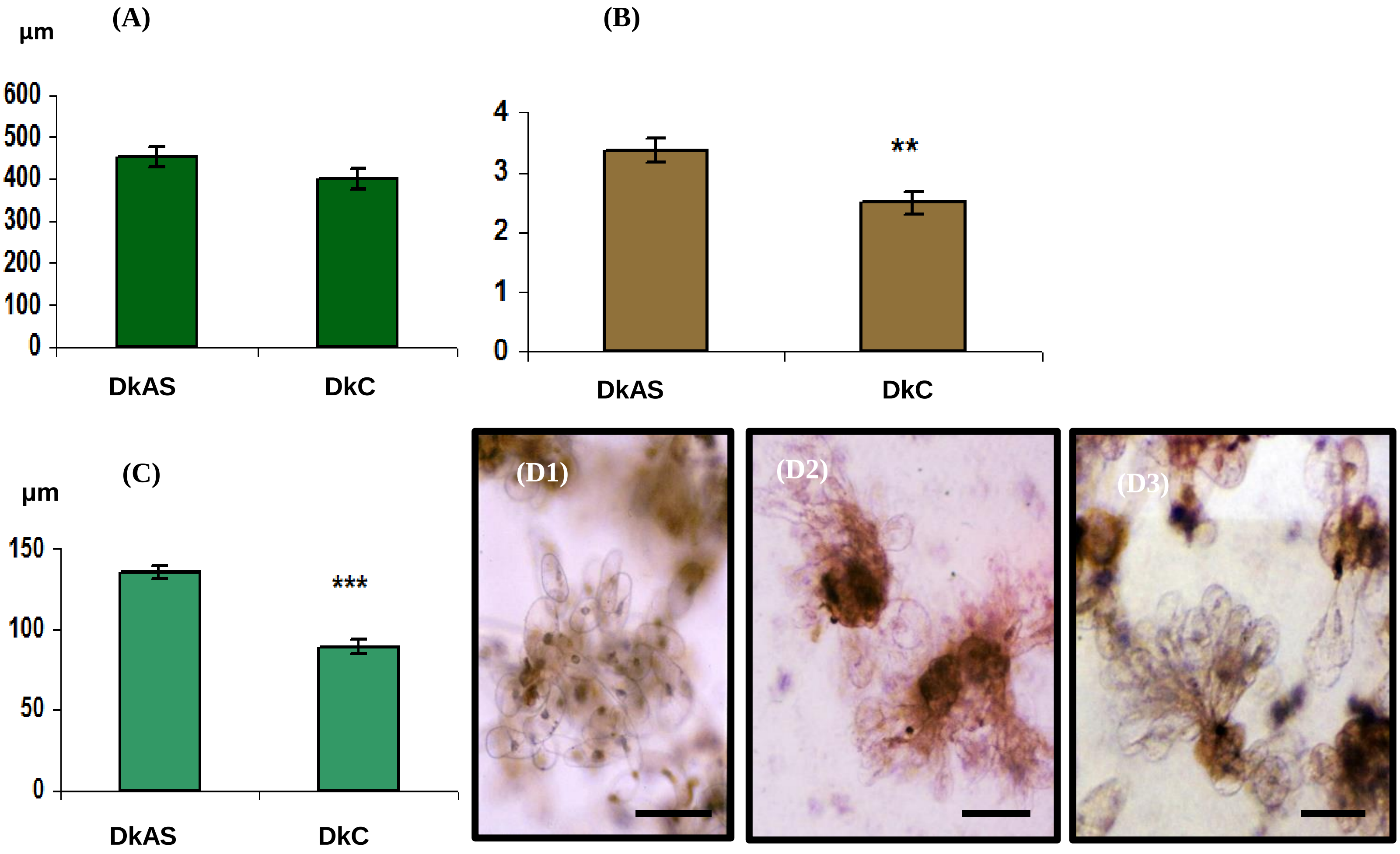

Supplementary Fig. S2 Growth of protoplast-derived cell colonies for the somatic hybrids (SH) *S. tuberosum* 'Delikat' + *S. chacoense* HL (*chc*) transformed with antisense *Atmsh2* gene (DkAS) in comparison to wild type somatic hybrids (DkC): **(A)** colony mean diameter (µm); **(B)** mean number of cells per colony; **(C)** mean cell diameter (µm); all measured at two weeks in culture ** significant at $p < 0.005$; *** significant at $p < 0.001$; **(D1)** cell colonies derived from potato cv. 'Delikat' mesophyll protoplasts; **(D2)** colonies from SH with wild type *chc*; **(D3)** colonies of SH involving *chc* AS (three weeks old). Scale Bar = 100 µm.
