# Supplementary material for: New Phenotypes of Potato Co-induced by Mismatch Repair Deficiency and Somatic Hybridization
Source: Front Plant Sci. 2019 Jan 22;10:3. doi: 10.3389/fpls.2019.00003 (PMC6349821; doi:10.3389/fpls.2019.00003)
Supplement: Supplementary file 8 [file Image_3.pdf]

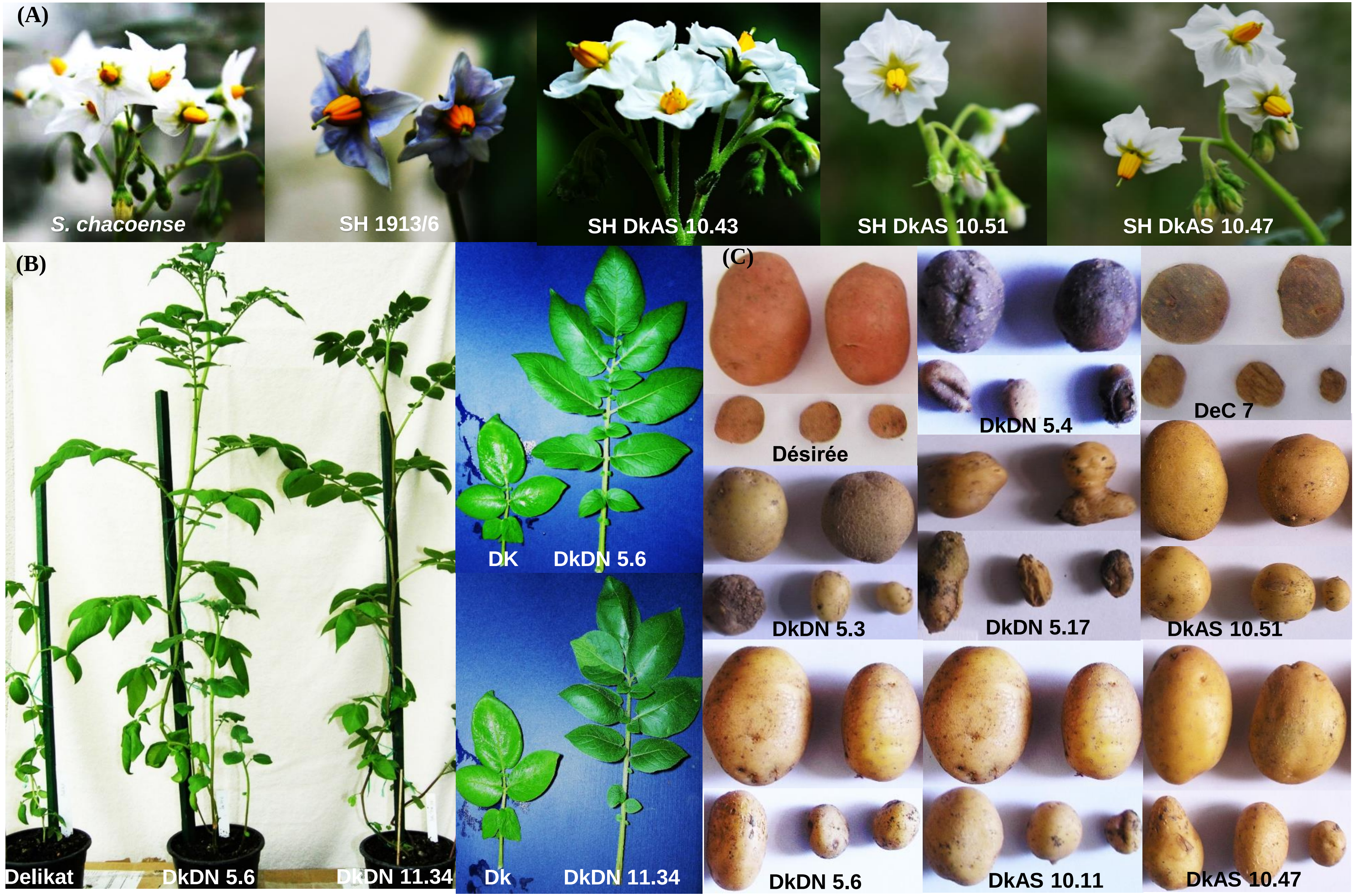

Supplementary Fig. S3 The phenotypes of parental species, selected wild type somatic hybrids and MMR deficient (AS or DN) somatic hybrids (SHs): **(A)** flowers, **(B)** whole plants and leaves; **(C)** tubers collected from greenhouse grown plants (the two biggest and the three smallest tubers are shown for each genotype). Note the purple flowers in wild type SH 1913/6 and purple or deformed tubers in DkDN 5.4 or DkDN 5.17, respectively.
