# Supplementary material for: New Phenotypes of Potato Co-induced by Mismatch Repair Deficiency and Somatic Hybridization
Source: Front Plant Sci. 2019 Jan 22;10:3. doi: 10.3389/fpls.2019.00003 (PMC6349821; doi:10.3389/fpls.2019.00003)
Supplement: Supplementary file 9 [file Image_4.pdf]

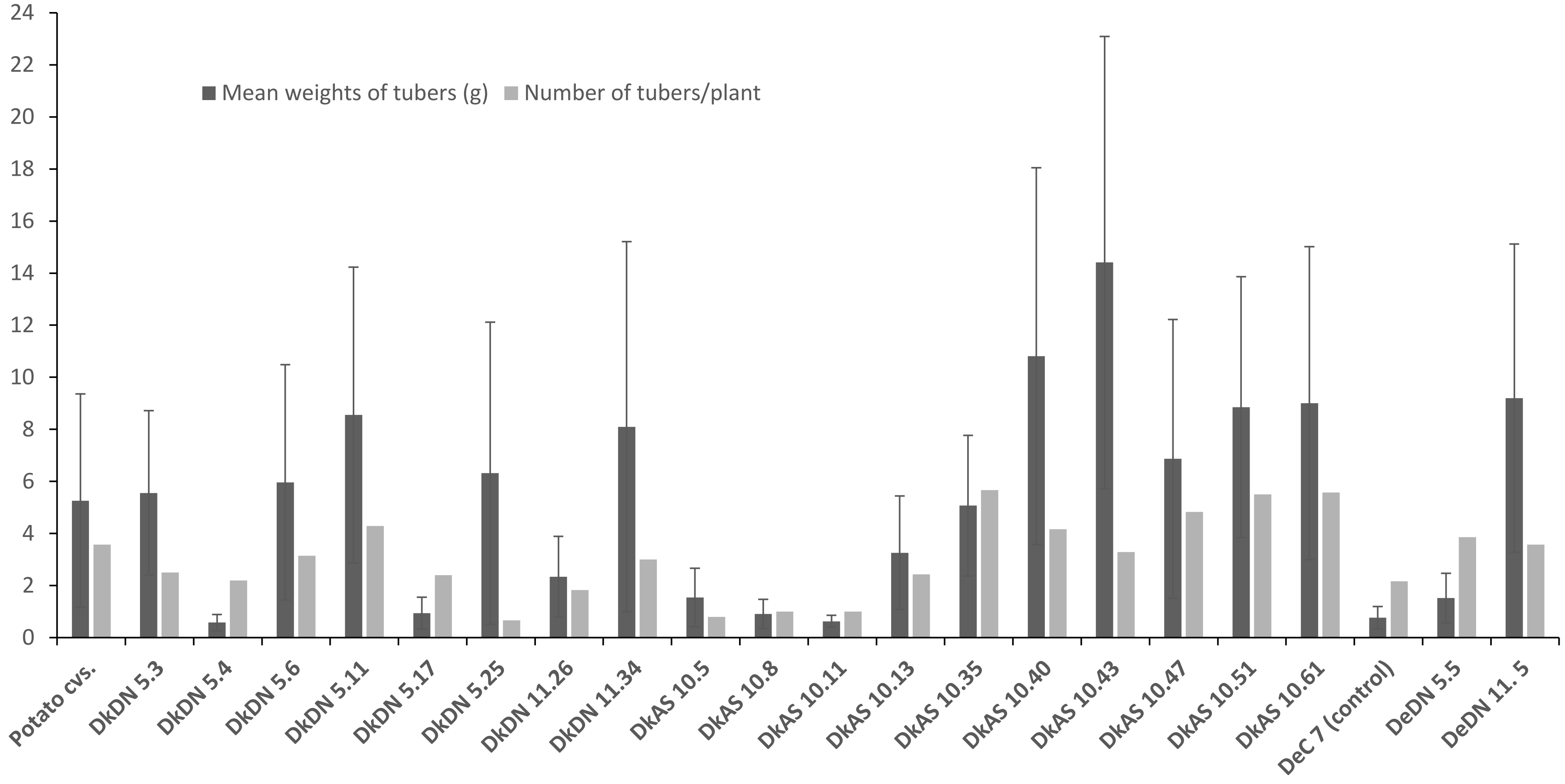

Supplementary Fig. S4 Mean weight and number of tubers in selected parent *Solanum tuberosum*, wild type somatic hybrid (SH) DeC 7 and MMR deficient (DN or AS) SHs harvested from greenhouse grown plants (n= 5); bars = SE
